# Spalt expression and the development of melanic color patterns in pierid butterflies

**DOI:** 10.1186/2041-9139-4-6

**Published:** 2013-02-19

**Authors:** Andrew M Stoehr, Joseph F Walker, Antónia Monteiro

**Affiliations:** 1 Department of Ecology and Evolutionary Biology, Yale University, CT 06511, New Haven, USA; 2 Department of Biological Sciences, Butler University, IN 46208, Indianapolis, USA; 3 Department of Botany and Plant Pathology, Purdue University, IN 47906, West Lafayette, USA; 4 Department of Ecology and Evolutionary Biology, Yale University, CT 06511, New Haven, USA

**Keywords:** Spalt, Butterfly wing patterns, Eyespots

## Abstract

**Background:**

Little is currently known about wing pattern development in the butterfly family Pieridae, which consists mostly of black melanized elements on white or yellow/orange backgrounds. A single transcription factor, Spalt (Sal), has been previously associated with the development of some pattern elements in *Pieris rapae*, but it is unclear to what extent Sal is associated with patterns in other pierid species.

**Results:**

We use immunohistochemistry targeting Sal proteins across several pierids and show that Sal is associated with dense patches of melanization across species but is not associated with vein-melanization or diffuse melanization on the wing. In addition, Sal is expressed along cross-veins and wing compartment midlines that do not develop melanization. Male and female *P*. *rapae* spots are sexually dimorphic in size and this dimorphism is also present in the domains of Sal expression. Finally, by disrupting cells positioned in the center of the anterior black spots of *P*. *rapae*, before and during the time of Sal expression, spot size was reduced.

**Conclusions:**

Our results suggest, but do not conclusively show, that pierid spots may develop in a manner similar to that of nymphalid eyespots, that is, containing a group of signaling cells at the center of the pattern responsible for the differentiation of the complete spot, and that spots and eyespots share at least one signal-response gene in common, the transcription factor Sal. We propose that focal differentiation and focal signaling mechanisms evolved prior to the split of the nymphalid and pierid lineages.

## Background

The complex and colorful wing patterns of butterflies are among the most conspicuous of animal traits, and as a result have a long history of study in the context of ecology and evolution [1,2]. More recently, butterfly wing patterns have proven to be highly amenable to research into the proximate – that is, genetic and developmental – mechanisms responsible for phenotypic variation [2-4]. Thus, butterflies are becoming ideal model systems for linking proximate and ultimate understanding of the evolution of animal forms.

Much recent work on the developmental genetics of butterfly wing patterns has focused on the so-called “eyespots” of members of the butterfly family Nymphalidae [5-11]. The nymphalid eyespot consists of a series of concentric rings of color in the adult butterfly and eyespot differentiation is associated with the expression of a number of different genes from late larvae to late pupal development that suggest a causal role in eyespot development [2,12].

Spalt (Sal) is the only transcription factor that, so far, spatially maps to both nymphalid eyespots and to adult wing patterns in a member of a different basal butterfly family, Pieridae. In the pierid butterfly *Pieris rapae* (the cabbage white), Sal is expressed during the early pupal stage in areas that later will correspond to black spots and wing tips in the adult [10]. These melanic pattern elements form much of the conspicuous and ecologically relevant phenotypic variation in the Pieridae [13,14]. For example, black patches have been implicated in inter-sexual communication [15-17] and scattered black scales at the base of the wing function in thermoregulation across pierids [13,18-20]. This thermoregulatory role is thought to underlie the evolution of adaptive phenotypic (seasonal) plasticity in pierid wing patterns [13,21,22].

To gain greater insight into the developmental genetics of pierid wing patterns, we investigated a) whether Sal is also associated with black pattern elements in other pierid butterflies, b) whether Sal expression correlates with known wing pattern sexual dimorphism in *P*. *rapae*, and c) the developmental mechanism underlying spot development in *P*. *rapae*.

To test Sal’s association with additional pattern elements across pierids we examined wing pattern elements in *P*. *oleracea* and *Colias* spp., and elements in *P*. *rapae* not previously explored in a previous study [10]. To test Sal’s association with sexually dimorphic wing patterns we examined the size of Sal expression domains in male and female *P*. *rapae*. In adults, the anterior spots are usually larger in females than in males, and the posterior spots in males are often entirely absent or are very reduced compared to the posterior spots of females [23]. Finally, we probed the underlying developmental mechanisms of spot production in *P*. *rapae* by inflicting wing damage to pupal wings at the center of the future spots (as well as at off-center locations), and by transplanting cells at the center of future spots in *P*. *rapae* to ectopic wing locations [24-27].

## Methods

### Animal husbandry

*P*. *rapae* specimens were captive-reared on collard greens (*Brassica oleracea*). *P*. *rapae* specimens were part of a lab colony originally derived from animals purchased from Carolina Biological Supply (Burlington, NC, USA). *P*. *oleracea* butterflies were the filial generations of parents originally collected in Vermont near Texas Falls. (*Pieris oleracea*, sometimes also referred to as *Pieris napi oleracea* Harris, is a member of a large Holartic *Pieris napi* species complex. Readers are referred to [28] for details on the systematics of this species complex.) *Colias* butterflies were reared on white clover (*Trifolium rupens*), and were offspring of adults collected in New Haven County, Connecticut. In this area, two species of *Colias*, *C*. *philodice and C*. *eurytheme*, as well as hybrids of the two species, can be collected. Our rearing cages likely contained both species, and possibly the hybrids. Because these species are very similar in appearance, particularly with respect to the melanization patterns, we did not attempt to distinguish the species.

All *Colias* specimens, and some of the *P*. *rapae* and *P*. *oleracea* specimens, were reared at 25°C on a 12L:12D photoperiod. Some *P. rapae* and *P. oleracea* were reared at 19°C and 12L:12D to try to increase the extent of melanization in some pattern elements in these species. *P*. *rapae* were sexed as pupae according to the presence (female) or absence (male) of a sex-specific suture on the genital plate [29]; F. Chew, personal communication].

### Immunohistochemistry

Approximate pupation times were determined from time-stamped photos using a time-lapse camera set to photograph late-instar larvae every 30 minutes. Pupal wings were removed at various time points following pupation, then fixed and stained with primary and secondary antibodies following the protocols of [7]. Wing age ranged from 12 to 30 hours post-pupation in animals reared at 25°C, and from 24 to approximately 55 hours post-pupation for animals reared at 19°C. Animals reared at the lower temperature take approximately double the time to develop. When possible, both right and left wings were stained, mounted and observed.

Three different anti-Sal primary antibodies were used over the course of the studies described here. Two of the antibodies (grown in rats and rabbits, and generously provided by Rosa Barrio) were previously described to target Sal in both *Drosophila* and butterflies [7,30], and the third antibody (grown in guinea pigs) was raised against the same Sal protein sequence from *Drosophila* that generated the first two antibodies [30] and generated staining patterns indistinguishable for the other two antibodies in *Bicyclus anynana* and *Pieris rapae*. The guinea pig antibody was made by Proteintech Group, Inc. (Chicago, IL, USA) against an amino acid sequence that combined the sequences of both peptides that were separately injected into rats and rabbits in the previous study [30]. Four guinea pigs were injected with this synthetic peptide in order to generate polyclonal antibodies. The rat anti-Sal antibody was used at a 1:1,000 dilution, the rabbit anti-Sal at 1:500 and the guinea pig antibody (GP66-1) at 1:10,000. The secondary antibodies, all used at 1:200, included goat anti-rat, goat anti-rabbit and goat anti-guinea pig (Alexa Fluor 594; Molecular Probes, Invitrogen, Grand Island, NY, USA). Wings were mounted under cover slips on glass slides in SlowFade Gold (Invitrogen) mounting media, sealed at the edges with nail polish. Wings were viewed under a Nikon Eclipse 90i fluorescent microscope (Nikon Instruments, Melville, NY, USA), and photographed with a QImaging Retiga Ex*i* digital camera (QImaging, Surrey, BC, Canada).

### Quantification of sexual dimorphism of Sal expression in *P*. *rapae*

The area of the Sal expression domain in the M3 and Cu2 wing compartments, where the two central spots are located, was measured using Object J software (http://simon.bio.uva.nl/objectj/index.html). If both left and right wings for an individual were mounted and photographed, we used the one that, upon visual inspection and without knowledge of the sex or rearing treatment, was deemed to be the better-quality mount. Because Sal was expressed in easily distinguished rows of scale-building cells, it was possible to quantify the area of the expression by manually tracing the minimum-area polygon that enclosed all Sal-expressing scale-building cells. We also measured the anterior-posterior width of the wing compartment as an index of overall wing size. (Overall wing or wing compartment size could not be quantified because often the wings had folds or tears.) A full factorial analysis of covariance was performed on the size of the Sal expression domain of each spot using temperature and sex as fixed factors and wing size (measured as wing compartment width) as a continuous covariate. JMP 9.0.2 (SAS Institute, Cary, NC, USA) software was used for the analysis.

### Wing piercing experiments

**Figure 1:**
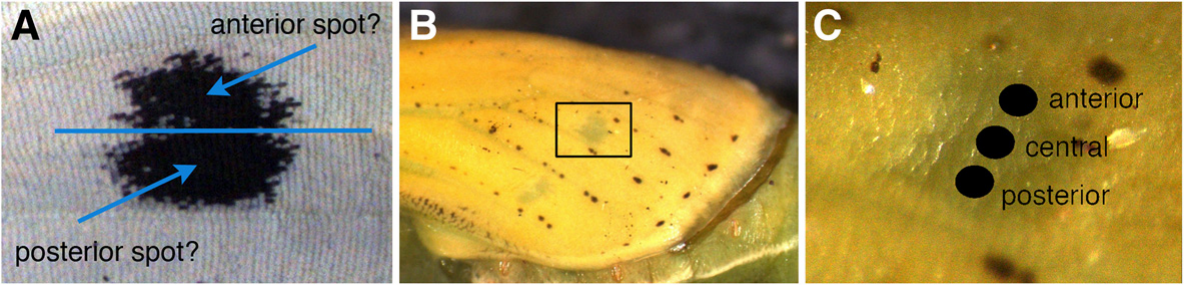
**One or two signaling centers? A**) The slight dumbbell-shaped *Pieris rapae* spot may have a single signaling group of cells at its center or two off-center signaling sources, one producing the anterior half of the spot, and the other the posterior half. **B**) Late *P*. *rapae* pupal wing showing the area that was targeted for piercing with underlying pigmented spot (the darker spot was not visible when operations were performed on younger pupal wings). **C**) The three different locations targeted for piercings on the young pupal wings.

We pierced the right wing of each *P*. *rapae* pupa at variable times post-pupation, from immediately following pupation to 35 h post-pupation. Wings were pierced using a glass needle made from a pulled capillary tube in one of three positions: as close to the center of the area where the M3 spot would later develop, and at off-center positions anterior and posterior to the center of the wing spot (Figure 1C). These off-center piercings were done because the spots are slightly dumbbell shaped (Figure 1A) and could potentially be the result of a fusion of two spots. If off-center damage leads to larger disruption of the spots than central damage, then this would support the fusion hypothesis. During the pupal stage, the wing veins are visible and the location of the position where the spot will later develop can accurately be determined based on slight but clearly visible wrinkles in the pupal cuticle in that area (Figure 1B). The piercing entered through the dorsal wing surface. After piercing, the animals were allowed to develop to eclosion. For this experiment *P*. *rapae* were reared at 23 to 25°C.

### Adult wing measurements

The right and left wings of the wing-pierced *P*. *rapae* were removed and the M3 spots were photographed in both wings. We used the image manipulation software GIMP (http://www.gimp.org) to overlay a grid with 8 columns and 10 rows on each control spot, so that the spot was perfectly contained within the grid. Then, this grid was overlaid and centered on the experimental spot along the horizontal axis, and centered vertically using the mid-line fold of the wing and the wing veins as guides. Both images with overlaid grids were then converted into black and white in Image J. Using the macro “Butterflies” (http://lepdata.org/monteiro/Protocols.html) each square of the 8 × 10 grid (80 squares) was re-drawn in a particular fixed order in both control and experimental wings. After application of a fixed brightness threshold, the area of black pixels detected on each square of the grid was calculated. The default threshold value of 40 was used because it appropriately separated the black from the white scales on the images. The average areas of black pigment for each square of the grid for both control and experimental spots were calculated. These averages, once assembled along two grids of 8 columns and 10 rows in Microsoft Excel 2007 (for Windows) (Microsoft, Redmond, WA, USA), were subtracted to produce a new “grid” with the differences between the experimental and control spots. This new grid was used to define a “heat-map” of the area differences. Cells in the grid were conditionally formatted so that blue color indicated minimum values and red maximum values.

### Tissue transplant experiments

To test whether cells at the center of the future spot patterns in *P*. *rapae* are inducing surrounding cells to differentiate black scales, we transplanted a small cluster of cells (a rectangle of cuticle and attached dorsal wing epidermis approximately 0.2 by 0.3 mm) from the putative center of the M3 spots during the early pupal stage (two to five hours post-pupation) into a more distal location in the adjacent Cu1 wing compartment (n = 36). The transplant was rotated 180° so that we could later identify these cells from surrounding cells using scale polarity as a marker. Individuals were placed in individual plastic cups and adults were photographed upon eclosion.

## Results

### Melanic pattern elements in pierid butterflies that correlate with Sal expression

**Figure 2:**
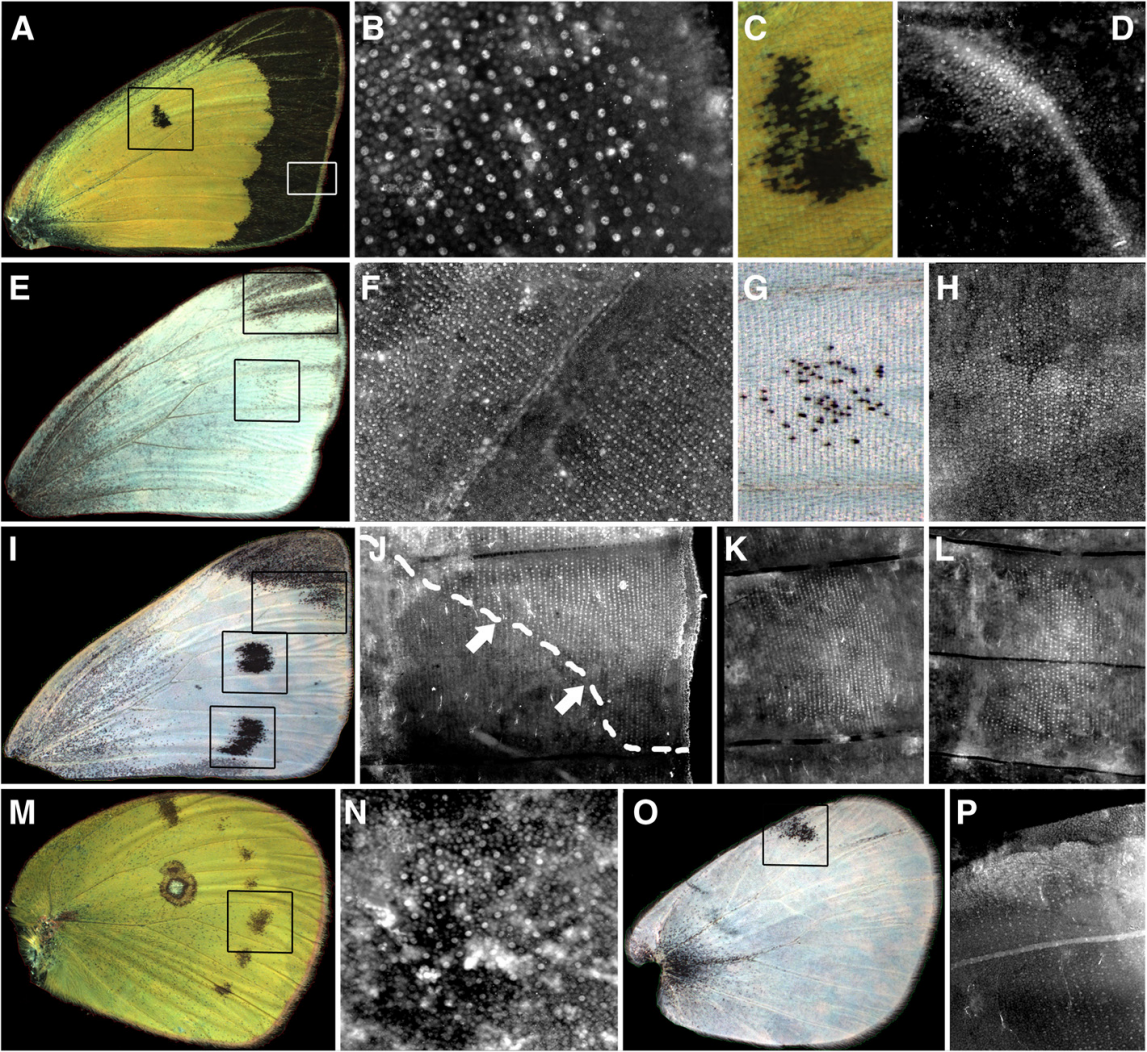
**Melanic pattern elements in pierid butterflies that correlate with Sal expression.** Wing surfaces depicted are dorsal forewings from (**A**) a male *Colias* spp.; (**E**) *P*. *oleracea*, sex unknown; (**I**) *P*. *rapae* female; (**M**) hindwing ventral surface of a male *Colias* spp.; and (**O**) dorsal hindwing of *P*. *rapae*. Insets indicate regions that are depicted at higher magnification to the right, in other panels. (**B**) Sal is expressed in scale-building cells (cells that show strong punctate expression and are arranged in regular rows) in the marginal region. The region where the cross-vein spot occurs in adults (**C**) also shows pupal expression of Sal in scale-building cell rows as well as in non-scale building cells (**D**) (24 h post-pupation; 25°C). (**F**) Sal is expressed in rows of scale-building cell at the wing tips of *P*. *oleracea* and in the (**G, H**) M3 wing spots (44 to 49.5 h post-pupation; 19°C). (**J**) Sal is expressed in rows of scale-building cells at the posterior margin of the wing-tip patch (arrows and dashed-line roughly indicate the border between Sal expression and lack of Sal expression, corresponding to the black-white transition of the adult wings), M3 spot (**K**) and Cu2 spot (**L**) (26.6 h post-pupation; 25°C). (**N**) Sal is expressed in rows of scale-building cells in the location where one of the spots will later develop (14 h post-pupation; 25°C). (**P**) Sal is expressed in rows of scale-building cells in the location where the spot (shown in **O**) will develop (23 h post-pupation; 25°C). Bright, non-regular spots on the wings correspond to non-specific staining or debris.

**Figure 3:**
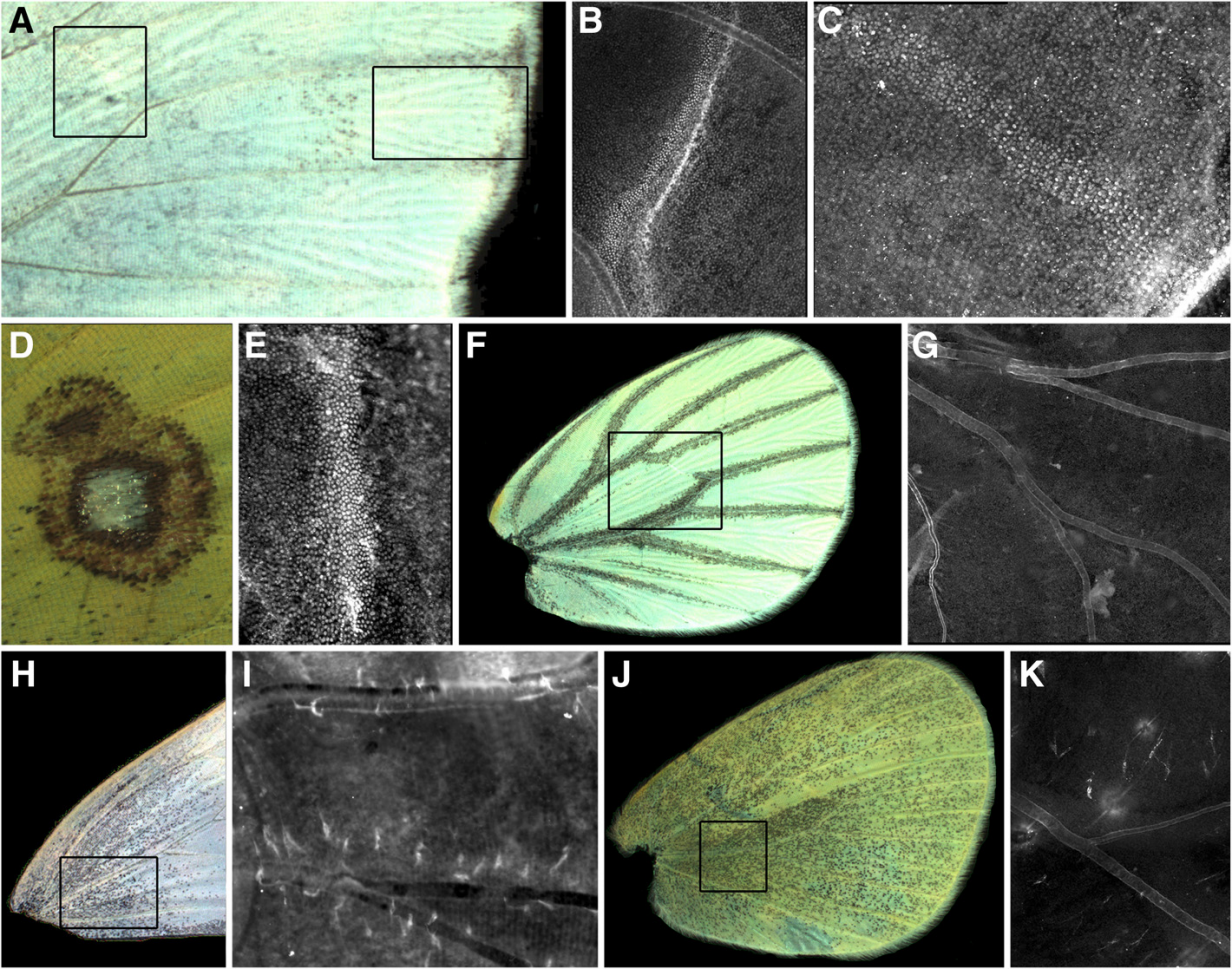
**Lack of correspondence between Sal and adult wing pattern elements.** (**A**) Close-up of *P*. *oleracea* dorsal forewing (as in Figure 1E), showing the lack of melanized pattern elements at the cross-vein region and wing margin. (**B**) Sal expression in the region of the cross-vein of *P*. *oleracea* but not in rows of scale-building cells. (**C**) Sal is expressed in the midline of a wing compartment extending proximally from distal wing margins; expression occurs in rows of scales as in the panels in Figure 1 (*P*. *oleracea*; 44 to 49.5 h post-pupation; 19°C). (**D**) Close-up of *Colias* ventral hind-wing cross-vein "eyespot". (**E**) Sal is expressed in densely-packed cells in the cross-vein, as in the forewing cross-vein, but not in rows of scale-building cells (12.5 h; 25°C). (**F**) Heavily-melanized veins of *P*. *oleracea* ventral hindwing lack detectable Sal expression (**G**) along these wing veins (44 h; 19°C). (**H**) Diffuse melanization of basal dorsal forewing in female *P*. *rapae* lack detectable Sal expression (**I**) (26.6 h; 25°C). (**J**) Diffuse melanization of ventral hindwing in *P*. *rapae* lack detectable Sal expression (**K**) in the ventral hindwing (23 h post-pupation; 25°C).

*Colias* butterflies show an extensive band of black, melanized scales along the distal margin of the forewing (Figure 2A; inset box in lower right corner) and hindwing (not shown). Sal is expressed in rows of scale-building cells in these same wing regions of the developing pupal wing (Figure 2B). The dorsal forewing also displays a small black spot over a crossvein in the middle of the wing (Figure 2A (center box), C). In the developing pupal wing, this area shows two patterns of Sal expression; a dense pattern of expression in cells that are not arranged in rows, in addition to expression in rows of scale-building cells (Figure 2D). The dense expression pattern appears not to be associated with later melanization in pierids as we also observed it in *P*. *oleracea* (Figure 3B) and *P*. *rapae* (not shown), which lack the black discal spot. Both *P*. *oleracea* and *P*. *rapae* show some melanization of the dorsal forewing tip (Figure 2E, I, respectively - see insets); we find Sal expressed in scale-building cells of developing pupae that map closely to these wingtip patterns (Figure 2F, J). Adult *P*. *oleracea* sometimes show a few melanized scales (Figure 2E, G) in the M3 wing compartment that, in *P*. *rapae*, shows a conspicuous spot (refer to Figure 2I for *P*. *rapae*). We found Sal expressed in scale-building cells in this wing region in a few *P*. *oleracea* specimens (Figure 2H), and we confirmed Sal expression in the region that maps to the wing spots of *P*. *rapae* (Figure 2K, L from [10], for comparative purposes). The ventral surface of the *Colias* hindwing has a series of black spots located centrally within each wing compartment (Figure 2M); Sal is expressed in rows of scale-building cells in these regions (Figure 2N). The dorsal hindwing surface of *P*. *rapae* contains a single black spot, located along the anterior edge of the wing (Figure 2O); this region also shows Sal expression (Figure 2P).

### Melanic pattern elements in pierid butterflies that do not correlate with Sal expression

Despite the correspondence between prominent black pattern elements and Sal expression in the pierid butterflies examined, not all instances of Sal expression are associated with melanization, nor are all adult melanic regions associated with pupal Sal expression. As mentioned above, in *Pieris* we found clear Sal expression at the discal cell cross-vein, though this expression did not occur in scale-building cells (Figure 3B). Unlike *Colias*, in *Pieris* there is no black pattern element in this region (Figure 3A, left inset). We also consistently found intervenous stripes of Sal expression, in both scale- and non-scale-building cells, extending from the wing margin into the proximal region of the wing compartments (Figure 3A, right inset; Figure 3C). These intervenous stripes are similar to those found for Distal-less, Notch and Sal, in a variety of butterfly species [9,10], and do not appear to be associated with any black pattern elements in the adult wing.

The discal cross-vein region of the *Colias* ventral hindwing contains a pattern element resembling two fused eyespots, one of these containing a central group of white scales surrounded by rings of colored scales (Figure 3D). Sal is expressed in this region in a dense line of cells (Figure 3E), as in the cross-vein region of the forewing, but we found no evidence for Sal expression in rows of scale-building cells in this region of the hindwing. The ventral hindwing surfaces of *P*. *oleracea* display wing vein melanization, particularly in animals reared at lower temperatures (Figure 3F); however, we never detected Sal expression along these wing veins. In *P*. *rapae*, a speckling of black scales are found in the basal portion of the dorsal forewing of females (Figure 3H) as well as on the ventral hindwing surface of both males and females (Figure 3J); these forms of melanization are often extensive in animals reared at lower temperatures. These wing regions also failed to show any Sal expression (Figure 3I, K). Our failure to detect Sal expression in *P*. *oleracea* wing vein regions and in *P*. *rapae* ventral hindwings and basal forewings occurred in animals raised at both rearing temperatures and in animals showing presence of Sal in other wing regions.

### Sexual dimorphism of Sal expression

In order to test whether Sal expression correlated with sexual dimorphism in spot size in *P*. *rapae*, we measured the size of the Sal expression domain for the M3 and Cu2 wing spots (see Figure 2K, L) of a number of *P*. *rapae* males and females that were sexed as pupae, and reared under either cool (19°C) or warm (25°C) conditions. The distributions of the expression domains of these spots were highly positively skewed, such that a fourth root transformation was used to obtain normally distributed data [31]. The expression domain of the M3 spot was 18.1% larger in females (N = 22) than in males (N = 26), though this difference was not statistically significant (F_1,40_ = 2.04; *P* = 0.16). In adults, the sexual dimorphism of the M3 spot is greatest for the dorsal surface; male M3 spots on the ventral surface are more similar in size to M3 spots of females. It is, therefore, possible that we did not detect differences in Sal expression for that spot because we did not distinguish between Sal expression on the two wing surfaces in developing pupae. There was also no significant difference in the size of the Sal expression domain for this spot between those butterflies reared at two different temperatures (F_1,40_ = 1.64; *P* = 0.21), nor did these two factors interact (Sex by Temperature Interaction F_1,40_ = 0.55; *P* = 0.46). Neither wing size nor any of the interactions between wing size and the other factors were statistically significant (all *P* >0.39).

The Sal expression domain for the Cu2 spot was significantly larger (70.2%) for females (N = 16) than for males (N = 24) (F_1,32_ = 6.24; *P* = 0.02). This difference was largely due to the fact that of the 24 males for which we measured this spot, we could not detect Sal expression for 9 (37.5%) of them, whereas only one of 16 (6.25%) females showed no Sal expression in this wing region. (The reduction in numbers from the analysis for the M3 spot was due to mounts where we could not confidently measure this spot). Neither temperature (F_1,32_ = 0.85, *P* = 0.36) nor sex by temperature interaction effects (F_1,32_ = 1.03, *P* = 0.32) were significant. Neither wing size nor any of the interactions between wing size and the other factors were statistically significant (all *P* >0.16).

### Wing piercings during development affect spot melanization in *Pieris rapae*

**Figure 4:**
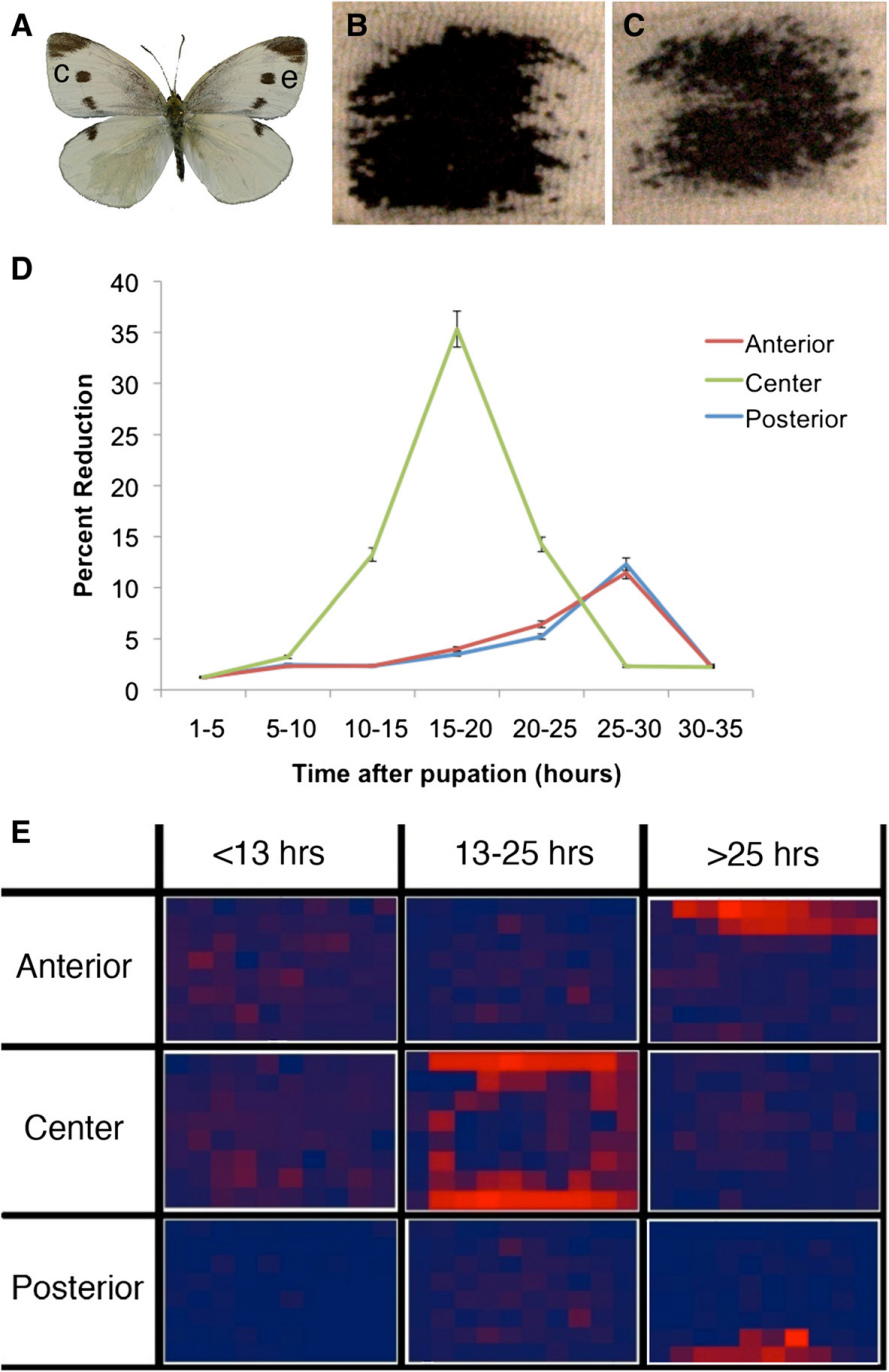
**Central piercing leads to large peripheral reductions in spot size in *P*. *rapae*. A**) Dorsal forewing surface of *Pieris rapae* showing the spots investigated in this study (e; experimental; c; control). **B**) Control, left wing spot. **C**) Experimental, right wing spot of the same individual after epidermal cell damage was inflicted at the center of the spot at 16 h post-pupation. **D**) Effects of wing damage on spot size reductions depend on position and timing of damage. Percent reductions (in area) were calculated relative to spot area on the control wing and averaged across individuals. Error bars represent 95% CI. **E**) Heat map of pigmentation changes in *P*. *rapae* anterior spots after wing damage at different spot locations and at different time periods after pupation (note larger time-interval binning relative to **D**). Pigmentation change is measured relative to the control spot in the same individual and averaged across individuals. Areas that show the greatest pigmentation change are in bright red and those that show the least change are in dark blue.

The right and left spots of unmanipulated wings are largely symmetrical (Figure 4A), thus, any substantive differences between right and left M3 spots can be attributed to our treatments. Only piercings applied to the center of the M3 spot in *P*. *rapae* led to substantive reductions in spot size, up to 35% of the spot area (Figure 4B-D); damage anterior or posterior to the developing spot had little if any effect until damage was applied late (after 25 h after pupation), when it led to a reduction in spot area of around 10% (Figure 4D; Table 1). The extent of spot damage depended on the timing of damage. Damage applied to the center from 15 to 20 h post-pupation led to a strong reduction in spot area, whereas damage applied earlier or later had a smaller effect (Figure 4D).

**Table 1:** **Number of individuals used for averages in Figure **4**D, using a five-hour bin size**

	**1 to 5 hours**	**5 to 10 hours**	**10 to 15 hours**	**15 to 20 hours**	**20 to 25 hours**	**25 to 30 hours**	**30 to 35 hours**
Anterior	2	3	7	5	3	4	4
Center	5	5	7	4	5	4	3
Posterior	2	3	4	8	4	5	4

In order to more precisely map where spot size reductions were taking place, we super-imposed a grid with a fixed shape and a fixed number of rows and columns on the two spots in each individual, where spot shape could be easily monitored. This allowed us to quantify whether damage led to pigment loss primarily in the areas where it was applied or away from these locations. Damage prior to 13 h post-pupation led to no effect on spot size, irrespective of position (Figure 4E; Table 2). Central spot damage, 13 to 25 h after pupation, led to reductions of black pigmentation along the periphery of the spot (Figure 4E). Damage to the anterior or the posterior halves of the spot, at the same time period, did not affect spot shape or size. Central damage after 25 h no longer affected spot size, whereas anterior or posterior damage at this time period led to pigment depletion restricted to the anterior or posterior edges of the spot, respectively (Figure 4E). These experiments suggest that cells at the center of the spot are primarily influencing spot size, and when these cells are damaged, spots become reduced at their periphery. Late damage to cells located anterior or posterior to the spot center also influence spot size, but these effects are expressed locally. Cells disrupted at these locations do not differentiate black pigment.

**Table 2:** **Number of individuals used to calculate the heat-map depicted in Figure **4**E**

	**0 to 13 hours**	**13 to 25 hours**	**25 to 35 hours**
Anterior	7	13	8
Center	12	14	7
Posterior	6	15	9

### Tissue at the center of the future spots *in P*. *rapae* contains a melanization factor

**Figure 5:**
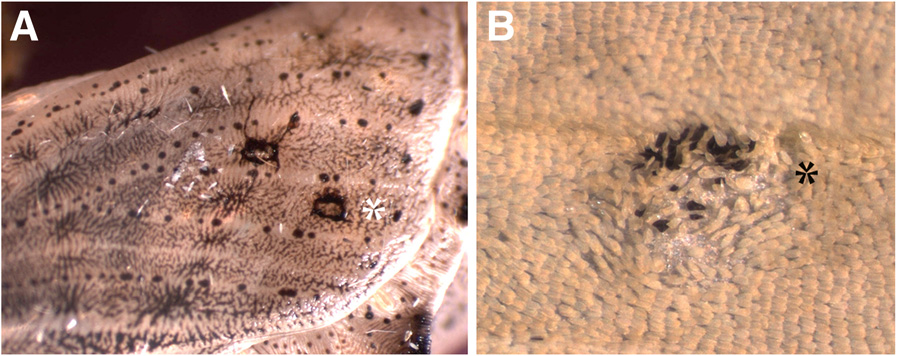
**Cells of the future M3 black spots contain a factor that differentiates black scales. A**) Operated *P. rapae* pupae where small rectangular pieces of cuticle attached to dorsal epidermis are exchanged from the area where the anterior spot develops into a more distal location on the wing (marked with *) at 2.5 h post-pupation. **B**) The adult wing pattern at the distal grafted site displays black scales with abnormal polarity resulting from the rotated graft.

Transplanted cells from the center of the future M3 spot in *P*. *rapae* contain a factor that leads to scale melanization. While most of the transplants yielded only white scales, five contained a cluster of black scales (Figure 5). None of the black scales, however, differentiated outside of the rotated transplant. This indicates that the factor that differentiates black scales is already present at the center of the future spots at approximately two hours post-pupation, but we were unable to show that this factor is a diffusible signal because no black cells differentiated outside the grafted piece of tissue.

## Discussion

### Sal expression and adult wing melanization patterns

We investigated the extent that the mainly “black and white” wing patterns in pierid butterflies were associated with the expression of a single transcription factor Sal, previously associated with black wing patterns in a single species of the group [10]. We discovered that a simple hypothesis that "Sal expression equals melanization, lack of Sal equals no melanization" is not tenable for pierid butterflies. Sal expression clearly maps to dense regions of melanization, such as the spots and wingtip patch of *Pieris* and the heavy wing margin melanization of *Colias*. However, the diffuse melanization at the base of the forewings and ventral hindwings of *Pieris* and the vein-dependent melanization patterns of *P*. *oleracea* are not pre-patterned by Sal. Conversely, Sal is expressed in regions that do not ultimately become melanized, such as along the cross-veins and in the midline of the *Pieris* species investigated here. We will address these different observations in turn both from a mechanistic as well as an evolutionary perspective.

### Melanized wing patterns, Sal expression and patterns of ecological covariances

The partial correspondence between Sal expression and adult melanization raises interesting questions about the development and evolution of these melanized traits. In particular, the Sal-associated and non-Sal-associated melanized wing patterns correspond to two previously identified sets of wing pattern elements in pierid butterflies showing positive covariance within a set but negative covariation across sets. For example, seasonal and geographical variation often results in positive correlations between the extent of melanization of the wingtip patch and spots of *Pieris* butterflies, but these pattern elements covary negatively with the basal diffuse melanization of the ventral hindwing and dorsal forewing, which in turn covary positively with each other [21,23]. Typically, these patterns of variation are related to the ecology of the butterflies: ventral hindwing and basal dorsal forewings tend to be more heavily melanized at colder geographical locations and/or during colder times of the year, whereas the spots and wingtip patches show the opposite pattern [21,23]. Previous researchers [21], based on studies of both phenotypic and genetic correlations, suggested that these forms of covariation reflected underlying developmentally homologous characters. Our findings for patterns of Sal expression lend support to this hypothesis. Pattern elements co-expressing Sal appear to be under similar regulatory control and respond similarly to environmental variation, whereas Sal-independent pattern elements appear to have a different underlying developmental basis and a different response to the same environmental stimuli. Our results suggest that some melanic wing pattern elements may require Sal expression to develop, while others do not.

### Sal expression and absence of adult melanization

Intervenous stripes of gene expression, including Sal expression, have been observed in a number of species, but these do not usually correlate with colored intervenous stripes in the adults [9,10]. Intervenous pattern elements are not very common in butterflies in general, and seem particularly rare in pierids, but a few species in the family (for example, some members of the genus *Leptophobia*) show clear melanized intervenous stripes. It is possible that in these species a pre-existent Sal pre-pattern may have become functionally connected to the melanin synthesis pathway, whereas in the currently studied species, this connection has not yet been made or has been interrupted during the course of evolution.

Sal expression is also correlated with the presence of a cross-vein during the early pupal stage in all pierids investigated here. This expression pattern has also been observed during the larval stage of *Pieris rapae* and in both larval and pupal stages of *Bicyclus anynana*, a nymphalid butterfly (A. Monteiro, J. Oliver, unpublished observations). There is, however, no melanized wing pattern associated with Sal and with cross-veins in these species. It is possible that Sal participates in cross-vein formation in a separate gene regulatory network from that involved in pigmentation. In *Drosophila*, Sal is involved in the early positioning of a longitudinal wing vein as a downstream direct target of the TGF-β signaling pathway ligand Dpp (reviewed in [32]), and while we are unaware of any evidence showing Sal expression in *Drosophila* cross-veins, TGF-β signaling is activated later in development along the fly’s cross-veins [33], suggesting that Sal may also be activated there, and that the cross-vein gene regulatory network may be conserved between flies and butterflies.

The hypothesis of a dual vein-development/coloration function for Sal in pierids is also supported by the fact that Sal appears to be expressed in distinct populations of cells associated with the cross-veins and with scale pigmentation. For example, *Colias* butterflies have a small melanized spot flanking the forewing crossvein, and there we see both kinds of Sal expression, that is, in rows of large scale-building cells as well as in more densely-packed putative future vein-cells of *Pieris* and *Bicyclus*.

On the ventral hindwing, *Colias* butterflies have an eyespot-like pattern, with a thin ring of melanized scales resembling nymphalid eyespot patterns [7], but surprisingly we observed no Sal expression in the rows of scale-building cells; expression was only present in the densely-packed vein cells. It is likely that a transcription factor other than Sal controls melanization in these scales.

### Sal expression and sexual dimorphism in *Pieris rapae* wing spots

The sexually dimorphic adult wing spots in *P*. *rapae* are pre-patterned by sexually dimorphic Sal expression patterns. This means that either Sal or its up-stream regulators are targets of sex-specific developmental regulation [34]. Interestingly, the sexes are also dimorphic for the diffuse melanization of the basal dorsal forewing [23], despite no apparent Sal expression in this wing region. This suggests that the different gene regulatory networks underlying these melanized wing patterns are both targets of sex-specific regulation.

### Spot signals, Sal expression and developmental mechanisms of spot formation

Both the wing damage experiments and the transplantation experiments in *P*. *rapae* suggest, but do not conclusively show, that a black-scale differentiating factor is present in the cells at the center of the future black spots in the M3 wing compartment as early as two hours post-pupation, and that this factor is likely a signal, broadly-defined, which diffuses or otherwise moves to surrounding cells. Obtaining pattern differentiation outside of a grafted cluster of cells is a critical piece of evidence to define the central cells as signaling cells [24,26]. The transplantation experiments, however, did not induce patterns outside of the transplanted tissue. It is possible that signaling from the transplanted tissue is weakened or delayed due to tissue healing, and central cells are not able to secrete sufficient signal to induce a spot beyond a few cells in diameter. The damage experiments, however, provided some support for a signal being produced at the spot’s center. Early damage to these central cells did not prevent them from recovering from the disruption and from producing a normal-sized spot. Slightly later damage, however, which produces large reductions in spot size, may be interpreted as disruptions to the production of sufficient signal before surrounding cells read and interpret this information. Off-center damage at this time, however, does not impact spot size, suggesting that damage to the central cells specifically, and not just damage to cells where black pigmentation will be produced, impacts spot development. Finally, later damage, when presumably most of the signal has been interpreted, no longer alters final spot size.

These experiments, which identify the location of putative signaling cells and the timing of signaling, are important first steps in identifying the actual signals using molecular techniques, such as *in situ* hybridization with candidate genes [10,35] or RNA microarray (or RNAseq) discovery approaches [36]. Future genetic knockdown studies, once putative signals have been identified, need to be performed in order to more thoroughly test the central signaling hypothesis proposed here. We propose, additionally, that the transcription factor Sal could be responding directly to a central signal because Sal expression occurs during the same time window (13 to 27 h) as signaling is taking place in *P*. *rapae*[10]. Sal is also known to be expressed concurrently with signals produced in the center of nymphalid butterfly eyespots [7,10].

Late damage, applied either anterior or posterior to the spot center, led to black pigment depletion at the site of damage. We propose that damage to these cells, some distance away from the central signaling cells and at a time when signaling appears to have ended (that is, when central damage no longer alters spot size), may interfere with the cell’s ability to interpret or translate the central signal and lead to melanization. Wound healing in pupal wing epidermal cells in *P*. *rapae* appears to involve de-differentiation of cells, in a more or less extensive region around the wound site, and cell growth from the margins of the wound [37]. This healing mechanism may prevent wounded tissue from being competent to receive the central signal or from being able to activate downstream components of the black scale differentiation gene network. None of these hypotheses, however, can readily explain why central damage at the same late time in development does not alter local (central) melanization. Further insight into the mechanisms that lead to peripheral pigmentation depletion in early-wounded wings, and to local pigmentation depletion in late-wounded wings, awaits further molecular characterization of the central signals, wound healing signals and their interaction in *P*. *rapae*.

## Conclusions

Schwanwitsch [38], and later Shapiro [39], both proposed similar hypotheses of pattern homologies across pierid species. The extensive marginal melanization of *Colias* butterflies was proposed to be homologous to the wingtip patch and spots of *Pieris* butterflies, as both were hypothesized to be variations on a theme of anterior-posterior bands. Our results suggest that the spots in *Pieris* are differentiated by a group of central signaling cells, and that these spots can become part of a continuous band if additional signaling centers are present along flanking wing compartments. Additional experimental work in *Colias* marginal bands, as well as in *Pieris* wing tips, will be necessary to support homologous developmental mechanisms across these pattern elements.

Schwanwitsch [38] also proposed a system of homologies across pierid and nymphalid wing patterns where nymphalid sub-marginal bands, and not eyespots, were homologous to pierid spots. Our results suggest that both pierid spots and eyespots have a similar central signaling mechanism in the early pupal stage [26] and share a putative signal response gene - Sal [7]. *Pieris* spots, however, do not express any of the genes found in nymphalid eyespot centers during the larval stage [10]. We propose that differentiation of the cells at the center of both spots and eyespots and signaling from these cells evolved prior to the split of the nymphalid and pierid lineages and requires genes that have yet to be identified. The more complex nymphalid eyespot gene network (reviewed in [2,40]) may have subsequently evolved by a mechanism of intercalary evolution (*sensu*[41]), where additional genes were co-opted to the central differentiated cells, before focal signaling in the pupal stage [40]. These nymphalid-specific genes may have aided in the evolution of eyespots from simpler spots, but this hypothesis awaits functional investigation.

## Abbreviations

Dpp: Decapentaplegic;Sal: Spalt;TGF-β: Transforming growth factor β

## Competing interests

The authors declare no competing interests.

## Authors' contributions

AS, JFW and AM conceived and designed the study, analyzed results and wrote the manuscript. JFW conducted the wing damage experiments and AM performed the tissue transplant experiment in *P*. *rapae*. AS conducted all other experiments. All authors read and approved the final manuscript.

## References

[B1] Boggs CL Watt WB Ehrlich PR Butterflies: Ecology and Evolution Taking Flight 2003 Chicago, Illinois: University of Chicago Press

[B2] Monteiro A Prudic KM Multiple approaches to study color pattern evolution in butterflies Trends Evol Biol 2010 2 e2

[B3] Joron M Frezal L Jones RT Chamberlain NL Lee SF Haag CR Whibley A Becuwe M Baxter SW Ferguson L Chromosomal rearrangements maintain a polymorphic supergene controlling butterfly mimicry Nature 2011 477 203 206 10.1038/nature10341 21841803 PMC3717454

[B4] Reed RD Papa R Martin A Hines HM Counterman BA Pardo-Diaz C Jiggins CD Chamberlain NL Kronforst MR Kronforst MR Chen R Optix drives the repeated convergent evolution of butterfly wing pattern mimicry Science 2011 333 1137 1141 10.1126/science.1208227 21778360

[B5] Carroll SB Gates J Keys DN Paddock SW Panganiban G Selegue JE Williams JA Pattern formation and eyespot determination in butterfly wings Science 1944 265 109 114 7912449 10.1126/science.7912449

[B6] Keys DN Lewis DL Selegue JE Pearson BJ Goodrich LV Johnson RL Gates J Scott MP Carroll SB Recruitment of a hedgehog regulatory circuit in butterfly eyespot evolution Science 1999 283 532 534 10.1126/science.283.5401.532 9915699

[B7] Brunetti CR Selegue JE Monteiro A French V Brakefield PM Carroll SB The generation and diversification of butterfly eyespot color patterns Curr Biol 2001 11 1578 1585 10.1016/S0960-9822(01)00502-4 11676917

[B8] Beldade P Brakefield PM Long AD Contribution of Distal-less to quantitative variation in butterfly eyespots Nature 2002 415 315 318 10.1038/415315a 11797007

[B9] Reed RD Serfas MS Butterfly wing pattern evolution is associated with changes in a Notch/Distal-less temporal pattern formation process Curr Biol 2004 14 1159 1166 10.1016/j.cub.2004.06.046 15242612

[B10] Monteiro A Glaser G Stockslager S Glansdorp N Ramos D Comparative insights into questions of lepidopteran wing pattern homology BMC Dev Biol 2006 6 52 10.1186/1471-213X-6-52 17090321 PMC1654149

[B11] Saenko SV Marialva MSP Beldade P Involvement of the conserved Hox gene Antennapedia in the development and evolution of a novel trait EvoDevo 2011 2 9 10.1186/2041-9139-2-9 21504568 PMC3108338

[B12] Nijhout HF Molecular and physiological basis of colour pattern formation Adv Insect Physiol 2010 38 219 265

[B13] Shapiro AM Seasonal polyphenism Evol Biol 1976 9 259 333

[B14] Nijhout HF The Development and Evolution of Butterfly Wing Patterns 1991 Washington, DC: Smithsonian Institution Press

[B15] Wiernasz DC Female choice and sexual selection of male wing melanin pattern in *Pieris occidentalis* (Lepidoptera) Evolution 1989 43 1672 1682 10.2307/2409383 28564326

[B16] Wiernasz DC Male choice on the basis of female melanin pattern in *Pieris* butterflies Anim Behav 1995 49 45 51 10.1016/0003-3472(95)80152-9

[B17] Ellers J Boggs CL The evolution of wing color: male mate choice opposes adaptive wing color divergence in *Colias* butterflies Evolution 2003 57 1100 1106 12836826 10.1111/j.0014-3820.2003.tb00319.x

[B18] Kingsolver JG Viability selection on seasonally polyphenic traits: wing melanin pattern in western white butterflies Evolution 1995 49 932 941 10.2307/2410415 28564878

[B19] Kingsolver JG Experimental manipulation of wing pigment pattern and survival in western white butterflies Am Nat 1996 147 296 306 10.1086/285852

[B20] Ellers J Boggs CL Functional ecological implications of intraspecific differences in wing melanization in *Colias* butterflies Biol J Linn Soc 2004 82 79 87 10.1111/j.1095-8312.2004.00319.x

[B21] Kingsolver JG Wiernasz DC Development, function, and the quantitative genetics of wing melanin in pattern in *Pieris* butterflies Evolution 1991 45 1480 1492 10.2307/2409894 28563829

[B22] Kingsolver JG Fitness consequences of seasonal polyphenism in western white butterflies Evolution 1995 49 942 954 10.2307/2410416 28564872

[B23] Stoehr AM Goux H Seasonal phenotypic plasticity of wing melanisation in the cabbage white butterfly, Pieris rapae L. (Lepidoptera: Pieridae). Ecol Entomol 2008 33 137 143 10.1111/j.1365-2311.2007.00931.x

[B24] Nijhout HF Pattern formation on lepidopteran wings: determination of an eyespot Dev Biol 1980 80 267 274 10.1016/0012-1606(80)90403-0 7004953

[B25] French V Brakefield PM The development of eyespot patterns on butterfly wings: morphogen sources or sinks? Development 1992 116 103 109

[B26] French V Brakefield PM Eyespot development on butterfly wings: the focal signal Dev Biol 1995 168 112 123 10.1006/dbio.1995.1065 7883067

[B27] Otaki JM Artificially induced changes of butterfly wing colour patterns: dynamic signal interactions in eyespot development Sci Rep 2011 1 111 22355628 10.1038/srep00111 PMC3216593

[B28] Chew FS Watt WB The green-veined white (*Pieris napi* L.), its Pierine relatives, and the systematics dilemmas of divergent character sets (Lepidoptera, Pieridae) Biol J Linn Soc 2006 88 413 435 10.1111/j.1095-8312.2006.00630.x

[B29] Genc H Determination of sex in pupae of *Phyciodes phaon* (Lepidoptera: Nymphalidae) Fla Entomol 2005 88 536 537 10.1653/0015-4040(2005)88[536:DOSIPO]2.0.CO;2

[B30] de Celis JF Barrio R Kafatos FC Regulation of the spalt/spalt-related gene complex and its function during sensory organ development in the *Drosophila* thorax Development 1999 126 2653 2662 10331977 10.1242/dev.126.12.2653

[B31] Quinn GP Keough MJ Experimental Design and Data Analysis for Biologists 2002 Cambridge: Cambridge University Press

[B32] Crozatier M Glise B Vincent A Patterns in evolution: veins of the *Drosophila* wing Trends Genet 2004 20 498 505 10.1016/j.tig.2004.07.013 15363904

[B33] Conley CA Silburn R Singer MA Ralston A Rohwer-Nutter D Olson DJ Gelbart W Blair SS Crossveinless 2 contains cysteine-rich domains and is required for high levels of BMP-like activity during the formation of the cross veins in *Drosophila* Development 2000 127 3947 3959 10952893 10.1242/dev.127.18.3947

[B34] Williams TM Carroll SB Genetic and molecular insights into the development and evolution of sexual dimorphism Nat Rev Genet 2009 10 797 804 19834484 10.1038/nrg2687

[B35] Werner T Koshikawa S Williams TM Carroll SB Chew FS Watt WB Generation of a novel wing colour pattern by the Wingless morphogen Nature 2010 464 1143 1149 10.1038/nature08896 20376004

[B36] Abzhanov A Kuo WP Hartmann C Grant BR Grant PR Tabin: The calmodulin pathway and evolution of elongated beak morphology in Darwin’s finches Nature 2006 442 563 567 10.1038/nature04843 16885984

[B37] Takayama E Yoshida A Color pattern formation on the wing of the butterfly Pieris rapae. 1. Cautery induced alteration of scale color and delay of arrangement formation Dev Growth Differ 1997 39 23 31 10.1046/j.1440-169X.1997.00004.x 9079032

[B38] Schwanwitsch B Wing pattern of pierid butterflies (Lepidoptera, Pieridae) Entomol Obozrenie 1956 35 285 301

[B39] Shapiro A Wohrmann K, Loeschke V The genetics of seasonal polyphenism and the evolution of “general purpose genotypes” in butterflies Population Biology and Evolution 1984 Berlin, Heidelberg: Springer 16 30

[B40] Oliver JC Tong X-T Gall LF Piel WH Monteiro A A single origin for nymphalid butterfly eyespots followed by widespread loss of associated gene expression PLoS Genet 2012 8 e1002893 10.1371/journal.pgen.1002893 22916033 PMC3420954

[B41] Gehring WJ Ikeo K Pax 6: mastering eye morphogenesis and eye evolution Trends Genet 1999 15 371 377 10.1016/S0168-9525(99)01776-X 10461206

